# The ethics and management of cannabis use in pregnancy following decriminalisation and licensing for medical use: narrative review

**DOI:** 10.1192/bjb.2021.102

**Published:** 2023-02

**Authors:** Abdulazeez Towobola, Basirat Towobola, Bosky Nair, Arti Makwana

**Affiliations:** 1 Kent and Medway NHS and Social Care Partnership Trust, Maidstone, Kent, UK; 2 East Sussex Healthcare NHS Trust, Hastings, East Sussex, UK; 3 Kent and Medway NHS and Social Care Partnership Trust, Maidstone, Kent, UK; 4 Kent and Medway NHS and Social Care Partnership Trust, Maidstone, Kent, UK

**Keywords:** Cannabis, pregnancy, risk, ethics, decriminalisation

## Abstract

**Aims and method:**

As drug policies pertaining to cannabis use become more liberalised, the prevalence of cannabis use in pregnancy could increase. However, there is limited guidance available for clinicians. This paper presents a narrative review of literature published in the past 16 years (2006–2021) to (a) address the impact of legalisation and decriminalisation on the risks, ethics and support of women who use cannabis during pregnancy and (b) develop guidance for clinicians.

**Results:**

Both national and international trends suggest increased use of cannabis over the past decade, while the risks of cannabis use for recreational or medicinal purposes in pregnancy remain unmitigated.

**Clinical implications:**

This review confirmed that the recommendation of cannabinoid-based products for pregnant and breast-feeding women is currently premature. More research is needed to address safety concerns. We discussed navigating ethical concerns and suggest targeted management strategies for clinicians treating pregnant women who choose to use cannabis.

The Crime Survey of England and Wales^1^ showed that in 2019 the most common drugs taken by adults aged 16–59 were: cannabis (7.6%; increased from 7.2% the previous year), powder cocaine (2.9%) and ecstasy (1.6%). Women in the reproductive years (18–44) were at the highest risk of developing substance use disorder;^1^ furthermore, 45% of pregnancies and one-third of births in England were unplanned and substance misuse was often identified as a risk factor for unplanned pregnancy.^2^ Almost one-third of drug users receiving substance misuse treatment were female, and over 90% of these women were of childbearing age (16–54 years).^3^ Trends in the USA paint a similar picture, with studies demonstrating an increased use of cannabis among both pregnant and non-pregnant reproductive-aged women^4^ and specifically among women during the first trimester of pregnancy.^5^ Indeed, a study examining national trends in the USA reported an increase of admissions to substance use treatment facilities for marijuana (cannabis) use during pregnancy from 29% in 1992 to 43% in 2012.^6^ It is clear that the use of cannabis has increased in the general population and in women of childbearing age; however, monitoring cannabis use during pregnancy remains difficult.

The prevalence of cannabis use in pregnancy varies widely and is largely affected by factors such as socioeconomic status, age and ethnicity.^7,8^ Sometimes, there is discrepancy between self-reported use and toxicology results. Self-reported rates of cannabis use are relatively low, whereas direct or objective measures of use often indicate higher rates.^9,10^ For example, one study found that out of 100 postpartum women, 11% disclosed cannabis use, whereas 14% tested positive by urinalysis and 28% by hair analysis.^7^ This discrepancy in reporting makes it difficult to fully ascertain the prevalence of cannabis use during the perinatal period and might indicate an underlying reluctance to disclose cannabis use which is likely to affect the quality of care and support provided to women who use cannabis during pregnancy.

Although current evidence indicates increasing prevalence of cannabis use, particularly in young women,^1^ this is an evolving field with limited guidance. This study is an up-to-date narrative review of literature to guide doctors and other clinicians who often find themselves in ethically, clinically and legally challenging situations in the management of pregnant women who use cannabis.^11^ We reviewed the ethics of cannabis use in pregnancy to give clinicians an evidence-based grounding in their principles of management, particularly when the dilemma of risks and benefits of cannabis use is heightened by advancements in medicinal cannabis, against the backdrop of previously known risks. Based on our findings, we recommend targeted interventions at different perinatal stages to contribute to policy discussions in local healthcare services and facilitate developing regional and national guidelines.

## Legalisation or decriminalisation

Legalisation refers to an approach that would remove all legal sanctions and penalties for possession and use of a drug. In this scenario, the government may establish regulations to manage licensing and to control the manufacture, quality, purity and supply of the drug, leading to it being more freely available to the general public (e.g. alcohol). In comparison, decriminalisation refers to an approach that would remove any criminal penalties despite it still being illegal to possess and use the drug (i.e. possession for personal use would not leading to a prosecution).^12,13^ Although decriminalisation of cannabis may seem to be an effective approach that recognises the potential harm and might be more favourably adopted by society, others claim that decriminalisation may only appear to be promising, as ‘a decriminalised drug is not legal, nor strictly illegal, depending on certain circumstances, nor is it necessarily of any better quality as it comes from the same sources as any other illegal drug’.^13^ Therefore the policy relies on authorities overlooking drug consumption and cannot adequality deal with the rising health concerns.

In light of the increasing evidence supporting the use of medicinal cannabis, there have been international calls for reform and review of drug policies on cannabis and cannabis-related products.^14^ Medicinal cannabis is currently legal in 33 states in the USA^15^ and countires such as Germany, Belgium, Spain, Italy, Australia and Canada.^14^ The oromucosal spray of cannabis (Sativex) for the treatment of multiple sclerosis was given regulatory approval in 30 countries.^14^ Changes in policy seem to meet public approval as there was a 5% increase to 48% in the public support for legalising cannabis, following the UK government legalising some medicinal cannabis products in 2018.^16^ Although the UK government legalised the use of medicinal cannabis,^17^ it retained the existing legislation for recreational use, listing cannabis as a Class B controlled drug under Part II, Schedule 2, of the Misuse of Drugs Act 1971 and Schedule 1 of the Misuse of Drugs Regulations 2001, stating that ‘it is unlawful to possess, supply, produce, import or export this drug except under a Home Office license’.^18^ Twenty-two percent of 1690 respondents in the YouGov survey said anyone should be allowed to cultivate their own drug.^16^

However, findings from international research have highlighted that decriminalising or legalising cannabis could lead to an increase in use, both among adults who have previously used cannabis and among those who may not have ordinarily used it (e.g. adolescents).^19–22^ For example, many states in the USA and some countries in Europe that have legalised cannabis show an increase in cannabis use and dependence, including among women of childbearing age.^23^ Although it is difficult to draw conclusions regarding the causal impact of decriminalisation on cannabis use (as trends are often different across age groups and across time),^20^ many factors, including perception of benefit and risk, increased drive towards autonomy, advances in pharmaceutical science and technology and financial motivation, have been identified as responsible for this wave of interest.^23–25^

Thus, liberalising cannabis policies may lead to an increase in cannabis use in the preconception, prenatal and postpartum period,^26,27^ but there is a clear lack of guidance available for clinicians working with women who may use cannabis during the perinatal period. It is critical to establish guidance, especially as recreational cannabis use in pregnancy can sometimes be associated with psychiatric comorbidity, disrupted parental care, and poor maternal and fetal outcomes^28^ (but see also^29,30^). Some use cannabis to manage nausea in pregnancy, albeit against medical advice,^31^ while conversely chronic use of cannabis has been associated with hyperemesis syndrome.^32^ Furthermore, there is a potential for medicinal cannabis to be used to treat menstrual disorders and menopausal symptoms, including hot flushes, anxiety and mood changes, insomnia, pain, reduced libido and fatigue,^33^ but there is limited research exploring the impact of medicinal cannabis use during pregnancy.^34^ Limitations in information available can affect the clinician's ability to make informed decisions to support care in this group. Clinicians may therefore find themselves in an ethically and clinically challenging position, balancing the health benefits of cannabis against the potential risks that may be incurred during pregnancy.

## Method and findings

We conducted a narrative review, performing searches on relevant databases (PubMed, MEDLINE, Embase, CINAHL, PsycInfo and Google Scholar) using the search terms: ‘cannabis’, ‘marijuana’, ‘tetrahydrocannabinol’, ‘THC’, ‘cannabidiol’, ‘CBD’, ‘cannabinoid’, ‘pregnancy’, ‘medical’, ‘medicinal’, ‘risk’, ‘ethics’, ‘management’, ‘legalisation’. All searches were limited by publication date (2006–2021). We identified 218 articles that met the search criteria; these were then screened and assessed independently by the authors for quality of evidence (e.g. large sample size, UK and international studies with multiple sites, studies that addressed confounders raised in limitations of previous studies) before inclusion in the review (111 articles). We excluded 107 articles with similar but no additional relevant information.

### Ethics approval

Ethical approval was not necessary for the research and writing of this review.

### Cannabinoid science

There are over 100 cannabinoids and other components in the cannabis plant, with tetrahydrocannabinol (THC) and cannabidiol (CBD) being the most studied.^35^ THC gives the recreational ‘high’ and CBD to some extent counteracts the psychoactive effect of THC.^35^ The phytocannabinoids bind to the endocannabinoid receptors CB_1_ and CB_2_, while also interacting with other neural transmission systems.^36–38^ CB_1_ and CB_2_ are G-protein-coupled receptors. CB_1_ receptors have psychoactive properties and are expressed in the central nervous system, gastrointestinal system, adipocytes, liver tissue and skeletal muscle. CB_2_ receptors, which are more restricted, are expressed in immune cells located in the tonsils, thymus, spleen and bone marrow, as well as in the enteric nervous system within the gastrointestinal tract.^39^ CB_1_ binding exerts its physiological and pathological effects by regulating presynaptic Ca^++^ levels, thus leading to a reduced release of neurotransmitters.^40^ At an early stage of human embryonic and fetal development (14 weeks), the endocannabinoid system develops in relation to other neurotransmission systems, and prenatal cannabis exposure could therefore lead to changes in activities of brain areas such as the prefrontal cortex, the mesolimbic system, the striatum and the hypothalamic–pituitary axis, with potential for long-lasting or delayed effects on executive function or regulation of the emotional system and cognition.^15,25,40^

### Medical use of cannabis

The earliest recorded date for cannabis use as a medicine is 4000 bce in China, and in the 19th and 20th centuries it has been used to treat migraine, neuropathic and musculoskeletal pain, and in childbirth.^41^ Following the discovery of the CB_1_ and CB_2_ cannabinoid receptors, research showing increasing medicinal properties has heightened interest in the legalisation of cannabis, specifically for medical use and to facilitate further research.^41^ Some formulations are currently used in treating resistant spasticity in multiple sclerosis, and nausea and vomiting induced by chemotherapy.^42^ CBD oral solution (Epidyolex^®^) has been classified as a Schedule 2 controlled drug in the UK for the treatment of seizures associated with Lennox–Gastaut and Dravet syndromes.^42^ Furthermore, in a recent randomised placebo-controlled phase 2 trial to establish the safety of dosing of CBD in non-pregnant individuals with cannabis use disorder, 400 and 800 mg appeared safe.^43^ In the UK, medicinal cannabis can be prescribed only by doctors on the General Medical Council's (GMC's) specialist register with special interest in the condition being treated, and under strict prescribing guidelines stipulated by the National Institute for Health and Care Excellence (NICE), GMC and NHS England.^44^

### Guidance on use of cannabis

Recent guidance from the UK's Food Standards Agency (FSA) on the increasingly popular oral non-medicinal CBD-containing products such as beverages (beer, spirits, wine, coffee and soda style drinks), oils (tinctures, drops, syrup, olive oils) and confectionary (gum drops, chocolate) limited their use to no more than 70 mg daily. It recommended that non-medicinal CBD products should not be used by pregnant or breastfeeding individuals, extrapolating from data on animal research which showed fetal harm.^45^ Likewise, in the USA, the US Food & Drug Administration (FDA) also highlighted the potential harm from cannabis (also referred to as marijuana) and although it approved the controlled use of medical cannabis (Epidyolex^®^), given the risks, which include serious liver injury, it strongly advises against the use of CBD, THC and marijuana in any form during pregnancy or while breastfeeding.^46,47^

Although guidance from official sources is relatively clear, there is a risk that information from other sources may provide conflicting advice to consumers. For example, the FDA reported that it has issued warning notices against three companies making unsubstantiated claims about their cannabis-based products’ ‘ability to limit, treat or cure cancer, neurodegenerative conditions, autoimmune diseases, opioid use disorder, and other serious diseases, without sufficient evidence and the legally required FDA approval’.^46^ Moreover, a study conducted in Colorado demonstrated that cannabis dispensaries often gave conflicting and sometimes incorrect information regarding the use of cannabis-based products during pregnancy. A mystery caller from the study team contacted 400 dispensaries stating that she was 8 weeks pregnant and was looking for advice regarding cannabis-based products for morning sickness. It was found that 35.7% of dispensaries endorsed the use of cannabis-based products during early pregnancy, 65% based their recommendation on personal opinion and only 31% recommended speaking to a healthcare provider without prompting.^48^ This demonstrates the importance of presenting a united front with official regulations and standards of advice, and of raising public health awareness regarding medical use of cannabis during pregnancy that is informed by scientific evidence.

### Cannabis and mental illness

Many people with psychological or mental disorders self-medicate with cannabis;^37^ however, the evidence on treatment efficacy is limited and often mixed.^49^ For example, a review of research into the use of CBD in treating fibromyalgia reported that, although findings seemingly suggested that CBD is a safe and effective treatment, the studies all suffered from methodological limitations that prevent firm conclusions being drawn.^50^ A recent systematic review and meta-analysis concluded that there is insufficient evidence that cannabinoids are effective in treating mental disorders, including depressive disorders and symptoms, anxiety disorders, attention-deficit hyperactivity disorder (ADHD), Tourette syndrome, post-traumatic stress disorder (PTSD) and psychosis.^51^ Another systematic review demonstrated tentative support for the efficacy of cannabis in social anxiety and as an adjunct in schizophrenia; however, the evidence in treating sleep disorders and PTSD was relatively weaker and there was no benefit of using cannabis to treat depression or mania.^52^ The authors concluded that the recommendation of cannabinoid-based interventions for psychiatric disorders would be premature at the moment and further warned against using products with high delta-9 THC content, particularly by young people.^52^ This is important to note as other studies have suggested that recreational cannabis with high THC content (e.g. skunk) is associated with mental health problems and physiological manifestations, including psychosis, dizziness, euphoria, drowsiness, dry mouth, confusion, somnolence and fatigue.^23,41,53^ It is clear that more research is required with human participants (rather than animals) to examine the safety of different strains and components, dosage and routes of administration.^41^

Advocates of medicinal cannabis suggest that it could reduce the rates of opioid dependence and deaths from overdose if patients switch from opioid-based pain relief to cannabis, as the risk of dependence is much lower and there are no reports of death from cannabis overdose.^41^ However, an international survey of 55 240 cannabis users published in 2020 found that the most commonly used classes of cannabis – sinsemilla and herbal (30.3%) and sinsemilla, herbal and hashish (20.4%) – were associated with increased dependence severity, while the class of concentrates and sinsemilla (1.7%) was associated with a record of diagnosed mental disorder.^54^ The conflicting evidence also highlights the need for further research.

### Impact of cannabis use in pregnancy

Factors associated with the continued use of cannabis during pregnancy include history of cannabis use disorder (2.77 times more likely to continue), frequent cannabis use (daily or weekly compared with monthly), not completing high school, having a psychiatric disorder, biological father's cannabis use and being unmarried.^6,55^ Personal (e.g. marital status, ethnicity) and area characteristics (e.g. urban, cosmopolitan or rural environments) as well as lifestyle factors can contribute to drug use; however, researchers warn that these factors often interact so it would be difficult to isolate the influence of any one of these characteristics on cannabis use.^1^

Studies have reported short-term risks for individuals who use CBD in pregnancy. This includes impaired short-term memory and motor coordination, altered judgement, paranoia, dependence disorder, psychosis, injuries, motor vehicle collisions and suicide.^53,56^ Indeed, maternal suicide remains the leading cause of direct deaths occurring within a year after the end of pregnancy;^57^ 8% of the women who died during or up to a year after pregnancy in the UK between 2016 and 2018 were at severe and multiple disadvantages, also known as the ‘toxic trio’ of mental health diagnosis, substance misuse and domestic abuse.^57^

Cannabis use in pregnancy has also been associated with still-birth, preterm labour, low birth weight, ‘small for gestational age’ and two-fold increased risk of admission to a neonatal intensive care unit.^58–60^ Gestational cannabis use may also be linked to maternal fatty liver, obesity, insulin resistance and increased risk of gestational diabetes mellitus (GDM).^61^ In a more recent study, prenatal cannabis use was associated with a 50% increased likelihood of low birth weight, independent of confounders, although no association was found for small for gestational age, preterm birth and neonatal intensive care unit admission.^62^ Furthermore, another recent population-based retrospective cohort study that included 661 617 pregnancies reported that cannabis users (*n* = 9427) were twice as likely to have a preterm birth compared with non-users, after adjusting for confounding variables, including tobacco use.^63^

Cannabis use can also affect breastfeeding as evidence suggests that THC and CBD can accumulate in breast milk.^64^ However, this remains a vastly understudied area. As THC is highly fat soluble it can be excreted into breast milk^65,66^ and released slowly over days to weeks, depending on extent of use. This is a concern as evidence suggests that breastfeeding mothers sometimes increase their consumption of cannabis after childbirth.^64^ Furthermore, cannabis use while breastfeeding appears to be associated with a decrease in infant motor development at 1 year of age.^67^ However, it is difficult to draw clear conclusions owing to small sample sizes and difficulty in separating the effect of exposure to cannabis *in utero* which continues into the postnatal period through lactation from exposure that occurs only during lactation. As studies (the majority of which are animal rather than human^68^) indicate conflicting outcomes, women who are unable to abstain are advised not to breastfeed within 1 h of inhaling or consuming cannabis, with the aim of reducing the infant's exposure to the highest concentration of cannabis in breast milk.^69^

The long-term effect of cannabis use should also be borne in mind as intrauterine exposure to cannabis has been found to increase the likelihood of initiation of cannabis and other substance use in adolescents.^70^ Prenatal exposure may also be linked to developing psychosis-like and affective disorders^71,72^ and ADHD.^24^ It is also linked with reduced attention and executive functioning skills, poorer academic achievement and behavioural problems.^73^ This might be because maternal prenatal cannabis use adversely affects growth of fetal and adolescent brains,^29,55^ as demonstrated in the reduction of inhibitory interneurons in the hippocampal formation of adult rats.^74^ In more serious cases, children raised in families where there is substance misuse can suffer negative effects on long-term health and well-being.^75^ Parents may be unable to supervise their children and meet their needs appropriately, which could lead to emotional abuse and neglect.^76^ There are further concerns about physical abuse of children if parents have difficulty controlling their own emotions. These children can suffer from behavioural, emotional and cognitive problems and experience long-term psychological effects from their experience of being raised in a chaotic household.^75^

Although research suggests that there is a high rate of abstinence from cannabis during pregnancy (with a discontinuation rate of around 77–78%),^77,78^ a high number of individuals also relapse, with 41% using cannabis within 3 months after delivery.^77^ Several characteristics could influence the rate of abstinence and relapse of cannabis use during the perinatal period. One study found that women with a post-secondary education and lower scores of depression and anxiety were more likely to attain abstinence, whereas those aged 21 years and below and with a diagnosis of depression were more likely to relapse in the postpartum period.^77^ Furthermore, given the high degree of concurrent substance misuse, women may substitute smoking cigarettes for alcohol, marijuana or cocaine use as they perceive cigarettes to be more socially acceptable and less harmful compared with illicit substances and alcohol.^77^ Thus, taking personal characteristics and the perception of harm into consideration is important for targeted perinatal interventions aimed at managing risks associated with cannabis use.

### Ethical considerations of drug use in pregnancy

The use of medicinal cannabis for different conditions has gained in popularity despite evident risks.^79^ Consequently, clinicians are often torn in the moral conflict and the debate around legalisation of medicinal and recreational cannabis use.^11,23^ Although legalisation and decriminalisation of cannabis across the world indicate a changing perception on the ethics of cannabis use, the stigma from the legacy of prohibition and ongoing restrictions are believed to stall research.^80,81^ However, the principles of beneficence and non-maleficence, respect for autonomy and justice, weighing costs and benefits, and patient-centred decision-making can all support evidence-based practice and can guide clinicians to recommend or prescribe cannabis when indicated.^80,82^

Claims that a fetus has full rights and that the right to life^83^ or prenatal care^84^ override the mother's right to autonomy or inviolability^85^ have led to jailing of women in the USA who took drugs in pregnancy or after birth if found positive on drug screening.^86^ It has been argued that criminalising substance misuse in pregnancy would cause more harm than good as the worry of penalisation may lead mothers away from adequate perinatal care and support for cannabis use disorder.^86^ By pitting the rights of the mother and fetus against each other, the discussion can easily become moralistic rather than evidence based and can perpetuate stigma associated with substance misuse during pregnancy. Furthermore, punitive measures place healthcare workers in difficult positions as they have also questioned the policy of drug screening in pregnancy without consent, their role in policing women who take cannabis in pregnancy and the disproportionate impact on women from minority ethnic groups.^86,87^

The principle of non-maleficence supports advising against cannabis use in pregnancy. However, for those who choose to use cannabis in pregnancy despite the warning, Bewley argues that as the fetus is a future member of society, society has a ‘legitimate interest’ in its welfare and should aim to support women who take drugs and minimise preventable harms to babies by offering incentives rather than using threats, coercion or punishment.^85^ Harris also argued in favour of a gender- and equality-based approach to resolving the dilemma rather than perpetuating the age-long conflict-based ethics.^11^ In this model, clinicians faced with ethical dilemmas should attempt to understand pregnant women and their decisions within their broad social networks and communities, ask how the clinician's personal standpoint influences outcomes judged to be ethical and determine whether the clinician's ethical formulations reduce or enhance existing gender, class or racial inequality.^11^ This seems to be a more appropriate solution as there is no evidence to suggest that penalising drug use in the perinatal period leads to better maternal or fetal outcomes.^86^ Bridging the gap between different views requires clarification of what constitutes benefits, harm and rights in relation to cannabis use as these are the core issues contended in the mother–fetus drug use debate.^83^

Proponents of recreational drug use contend that the government should intervene only when there is a high risk of causing harm to others.^88^ Prohibitionary laws based on harm reduction have been challenged as punitive^89^ and it has been proposed instead that harm reduction should be based on four principles: (a) drug use should be viewed neutrally, not moralistically; (b) a drug user is a citizen and member of a community, not a deviant individual or only an object of measures; (c) drug policy should be based on practice and science, not on ideologies and dogmatism; and (d) drug policy should respect human rights and support justice, not trample on them in the name of a ‘war on drugs’ or the goal of a drug-free society.^90^ These principles may also be useful in the context of healthcare, in that clinicians should ensure that women who use cannabis during pregnancy are treated in a neutral, non-stigmatising way and are supported to make healthcare decisions based on evidence, rather than ideology.

Table 1 shows ethical considerations and recommended approaches to navigating ethical dilemmas of cannabis use in pregnancy.

**Table 1:** Ethical principles and recommended approaches in navigating ethical dilemmas concerning cannabis use in pregnancy

Ethical principle	Considered approach
Beneficence	Discuss benefits and recommend cannabis-based medicinal products when indicated based on evidence
Non-maleficence	Discuss short-term and long-term risks to the mother, fetus and infant or child
Respect for autonomy	Assess whether the patient has capacity; obtain informed consent for procedures; demonstrate weighing of costs and benefits in collaboration with the patient- and patient-centred decision-making
Justice	Ensure adequate access to treatment and care irrespective of decision to take cannabis-based medicinal products

### Implications for doctors and other clinicians

It is important for doctors and other healthcare practitioners involved in supporting women of childbearing age to be aware of the benefits of medicinal cannabis and potential risks of cannabis use in pregnancy.^91^ This helps in discussing potential risks with patients and supporting them to make informed decisions.^24,80^ With the legalisation of medicinal cannabis^41^ and ongoing research into other areas of potential benefit, it is perhaps more challenging to correct the common perception that cannabis is relatively harmless.^25^ For example, in a survey of 51 healthcare providers to pregnant women in the USA, the providers (2nd–4th year obstetrics residents, nurse midwives and practitioners and faculty physicians) reported that they perceive cannabis use in pregnancy as less dangerous than use of other illicit drugs, while expressing the view that many pregnant women do not perceive cannabis as a drug.^92^ The perception of low risk associated with cannabis use has also been reported among young people,^24^ and more research is required to ascertain the effects of parental cannabis use on parenting and child and adolescent development.^71,93^ Table 2 provides a summary of interventions at different perinatal stages. Some of these interventions are targeted at vulnerable women or at-risk groups, whereas others are indicated for public health.

**Table 2:** Indicated or targeted interventions by healthcare professional at different perinatal stages

Perinatal stage	Indicated or targeted intervention
**Prenatal**	Provide psychoeducation, assess motivation to change and discuss risk–benefit analysis to enable patient to make an informed decision about cannabis use. Use opportunities to embed contraception and precontraception care discussions during health and non-health contacts for women of childbearing age. Help women to maximise health and well-being before pregnancy by improving lifestyle and diet. Targeted intervention and support (referral to liaison or community psychiatry and/or substance misuse clinic) for high-risk vulnerable women with history of cannabis use disorder, frequent cannabis use, concurrent tobacco or other substance use, and history of psychiatric disorder. Identify and attempt to address social problems that may lead to low levels of happiness, which have been associated with drug use. Particular attention should be paid to identifying determinants of social inequalities so as to consider referral for social support. Public health interventions and multimedia campaigns to educate women of childbearing age-group regarding risks associated with cannabis use.
**Antenatal**	The following factors should be noted in booking clinic and follow-up as they are associated with continued use of cannabis during pregnancy: history of cannabis use disorder, frequent cannabis use (daily or weekly compared with monthly), level of education, having a psychiatric disorder, biological father's cannabis use, and marital status. History of oral consumption of non-medicinal CBD-containing foods or products should also be noted. Multiple urine screens should be offered throughout pregnancy owing to underreporting in clinical interviews; reassurance may need to be given that this is not punitive but meant to identify those requiring support. Look out for cannabis withdrawal syndrome (CWS) in regular and dependent users – symptoms from three of the following groups within 7 days of stopping cannabis: irritability, anger or aggression; nervousness or anxiety; sleep disturbance; appetite or weight disturbance; restlessness; depressed mood; somatic symptoms such as headaches, sweating, nausea, vomiting or abdominal pain Provision of integrated clinics for pregnant women which offer a ‘one-stop shop’ for antenatal care, primary care, counselling services for substance misuse, parenting and social care support. Use of evidence-based treatment such as motivational enhancement therapy and cognitive–behavioural therapy with abstinence-based incentives for the treatment of cannabis use disorders.
**Postnatal**	A multidisciplinary approach involving the general practitioner, midwife, obstetrician, paediatrician and social worker is important, so clear communication between all healthcare professionals involved in the care of the patient is necessary. Referral to liaison or community psychiatry and/or substance misuse professionals may be required. Monitoring of withdrawal symptoms in newborn by paediatrician. Cannabis use is not recommended while breastfeeding, based on current evidence, but if a nursing mother wants to use cannabis, support should be provided in line with the ethical principles discussed above regarding the use of cannabis-based products for medicinal or recreational use.

### Comprehensive assessment within a multidisciplinary framework

We emphasise the importance of a multidisciplinary approach within health and social services because of the impact of substance misuse on the fetus, the mother and the newborn child.^94^ We also advocate supporting women at risk to maximise their health and well-being before pregnancy by improving lifestyle and diet. Accurate identification of cases, use of motivational interviewing techniques and access to cognitive–behavioural therapy (CBT) are vital.^28^ Comprehensive assessment, the cornerstone of management, should consider the risk to the physical and mental health of the mother during pregnancy, and ongoing childcare and parenting issues. This should lead to a plan and care package involving well-coordinated, multidisciplinary care with a specialist drug service working collaboratively with the general practitioner (GP), midwife, obstetrician, paediatrician and social worker.^94^

GPs’ ongoing relationship with patients enables them to establish rapport in order to discuss preconception health and promote positive changes in health and lifestyle. The World Health Organization (WHO) recommends the use of interventions that are brief, structured and require easy-to-administer tools. These interventions include information and motivational components that have been effective in primary care settings.^81,95^ Furthermore, a Cochrane review found that a combination of motivational enhancement therapy (MET) and CBT with abstinence-based incentives had stronger evidence in the treatment of cannabis use disorder compared with drug counselling, social support, relapse prevention and mindfulness meditation.^96^

Identifying cannabis withdrawal syndrome (CWS)^97^ in regular and dependent users is important as it can confound mental illness or early signs and symptoms of pregnancy such as nausea, headache and fatigue, which is why some patients present late for treatment of mental illness or for antenatal care.^94^ The DSM-5 diagnosis of CWS requires at least three of the following seven groups of symptoms developing within 7 days of reduced cannabis use: irritability, anger or aggression; nervousness or anxiety; sleep disturbance; appetite or weight disturbance; restlessness; depressed mood; and somatic symptoms such as headaches, sweating, nausea, vomiting or abdominal pain.^98^ This underscores the importance of training for healthcare workers in identifying symptoms and signs of cannabis misuse, particularly in techniques to improve the accuracy of obtaining a history of cannabis misuse in pregnancy.^99^

Women may hesitate to disclose cannabis use owing to fear of judgement and concerns that their parenting ability may be questioned. Day & George^94^ described the difficulty of accurately reporting the prevalence of high-risk drug use in pregnancy owing to underreporting resulting from ‘feelings of shame, denial and stigma experienced by the drug user, lack of awareness among professionals in antenatal services, the presence of comorbid psychiatric disorders, and sociocultural barriers that may prevent a thorough assessment’. Asking about type, quantity and impact of substance use by a trained professional is vital in evaluating risk. Wilson and colleagues have suggested questions to consider pertaining to substance misuse in the preconception or perinatal period. These should focus on empathic enquiry to assess the type and number of substances used, care and support available, impact on daily life and potential motivators for change.^100^ Roncero and colleagues advocate harm minimisation strategies by early detection and alerting pregnant women to the risks of cannabis use to themselves and the unborn baby.^24^ Owing to the likely underestimation from self-report (which may be linked to guilt, fear of legal consequences, stigma and social service referral), multiple urine screens during pregnancy may be a more reliable method to assess and monitor exposure to substances.^24^ Urine toxicology in a group of pregnant females identified twice as many cannabis users compared with self-report (4.9 *v*. 2.5%).^101^ Maternal hair and the meconium of the newborn have also shown higher detection rates of cannabis use compared with clinical interviews.^102^ The collection window for urine toxicology is 24–48 h (longer in regular consumers of cannabis), whereas maternal hair provides the advantage of giving information on the presence of THC over several months, given that 1 month corresponds to roughly 1 cm of hair growth from the scalp; however, testing is limited by hair type, hair products, amount collected and processing. Meconium cannabinoid testing is more expensive and is limited by the narrow collection window of 72 h.^103^

It is also important to ask patients about their consumption of non-medicinal CBD-containing foods or products, because of potential adverse effects. As stated earlier, there is limited research on the effects of CBD on embryonic development; however, several animal studies suggest that CBD can cause fetal harm.^104^ More specifically, animal studies on rhesus monkeys, dogs and rats showed that CBD exposure resulted in problems such as hepatoxicity, immunotoxicity, reproductive toxicity, changes to organ weights (i.e. increase in weight of liver, kidneys, heart, thyroid, thymus, spleen and adrenal glands and decrease in testicular weight) and alterations to cytochrome P450 drug-metabolising enzymes.^104^ Obtaining relevant information during prenatal or antenatal reviews therefore helps to target interventions using a person-centred approach.^24^ Finally, the high rate of relapse of cannabis use 3 months after delivery indicates that ongoing support should be made available to susceptible women and their infants in the postnatal period.^77^

Cannabis use during breastfeeding is a key dilemma for clinicians as the benefits of breastfeeding are very well known but early evidence indicates that CBD and THC can be transferred to the infant via breast milk.^65^ Current guidelines recommend abstaining from cannabis use during breastfeeding,^105^ but clinicians may still be conflicted between encouraging breastfeeding for the benefit of the child versus encouraging abstinence from cannabis-based products despite the potential medicinal benefits for the mother.^106^ Although research into cannabis use during breastfeeding is in its infancy, it is clear that a system-wide, multidisciplinary approach is needed to inform nursing mothers about the effects of cannabis on infants.^107^ For example, pharmacists and lactation consultants are in unique positions and may be able to identify those who are using cannabis, offer advice on abstinence, support with education on the known risks and benefits of cannabis use during breastfeeding and provide referrals for treatment, if required.^108,109^

## Recommendations and conclusions

Although there may be an increasing trend in the use of medicinal cannabis^110^ both among those prescribed it for medical conditions and those self-medicating because of perceived efficacy in managing their emotional and mental symptoms, more research is needed (in human rather than animal studies) to determine the safety of different strains and components, dosage and routes of administration, effect sizes for clinical outcomes and comparisons with existing treatments. Legalisation or decriminalisation may lead to the increased use of cannabis in pregnancy despite persisting risks. Clinicians should adopt a harm minimisation strategy when navigating the dilemma of the rights of the fetus versus those of the mother.

Pregnancy offers a ‘window of opportunity’ to identify and treat substance misuse in this vulnerable group along with other psychosocial problems associated with substance misuse in pregnancy.^27^ From our research, we have presented a stepwise approach to providing care and intervention targeting the prenatal, antenatal and postnatal stages detailed in Table 1.

Doctors treating pregnant women need to keep their knowledge up to date and be aware of the impact of cannabis use in the perinatal period, especially as studies show that cannabis use is underreported. This will enable the provision of targeted intervention and support, including adequate information for pregnant women to make an informed decision about cannabis use.

### Limitations

The findings of this review should be seen in light of some limitations. This paper is a narrative review aiming to offer an objective analysis in the current context of decriminalisation and licensing of cannabis for medical use and its ethical considerations during pregnancy. A systematic review with rigorous methodological approaches, considering new and emerging evidence assessing the impact of licensing of medicinal cannabis in the UK, may be able to draw more robust conclusions on this topic of significant public health interest.

## Data Availability

The authors confirm that the data supporting the findings of this study are available within the article, and supplementary materials (methods, literature search) are available from the corresponding author, A.T., upon reasonable request.

## About the authors

**Abdulazeez Towobola**, MBChB, MRCPsych, MA, is a consultant psychiatrist with Kent and Medway NHS and Social Care Partnership Trust, UK. **Basirat Towobola**, MBChB, MRCOG, is a consultant obstetrician and gynaecologist with East Sussex Healthcare NHS Trust, UK. **Bosky Nair**, MBBS, MRCPsych, MSc, PG Dip HCL, is a consultant perinatal psychiatrist with Kent and Medway NHS and Social Care Partnership Trust, UK. **Arti Makwana**, MSc, is a research facilitator with Kent and Medway NHS and Social Care Partnership Trust, UK.
